# Understanding the Mechanism of Social Attachment Role in Social Media: A Qualitative Analysis

**DOI:** 10.3389/fpsyg.2021.720880

**Published:** 2021-08-06

**Authors:** MaoSheng Yang, WenSong Zhang, Athapol Ruangkanjanases, Yue Zhang

**Affiliations:** ^1^School of Economics and Management, Beijing Jiaotong University, Beijing, China; ^2^Chulalongkorn Business School, Chulalongkorn University, Bangkok, Thailand

**Keywords:** attachment theory, social media, grounded theory, social attachment, qualitative analysis

## Abstract

Qualitative research method was used to explore the formation and development of the attachment relationship between users and social media in the process of using social media. Based on the attachment theory, this study selected three representative social media platforms, namely, TikTok, WeChat, and MicroBlog, as theoretical samples, and this study adopted NVivo12.0 to root, theorize, and construct the original data. Research shows that users are stimulated by co-creation value to stimulate changes in their psychological needs and self-expression, leading to the formation of social attachment. Among them, user participation is a prerequisite for driving the occurrence of co-creation value, creating a continuous-use scenario for the attachment relationship between individuals and social media. Further, psychological needs and self-expression play mediating roles between co-creation of value and social attachment and promote the occurrence of personal belonging to software platforms. The findings of this research better our understandings about the mechanism of developing social attachment from continuous use of social media and offer practical implications for commercial uses of social media platforms.

## Introduction

The attachment relationship between users and social media has attracted deeply from practitioners and scholars. Social media has nowadays played a decisive role in human progress and daily life of people. Throughout history, in the relevant fields of economic, social, and political development, the interaction between humans and social media results in many potential applications, which not only strengthens economic progress, but also becomes a powerful tool for human development (Ahmed et al., 2019; Lemay et al., 2019; Chen et al., 2020). However, the low conversion cost of social media during the interaction process poses a challenge for users to attach to the continuous use of social media and hinders the positive impact and progress of social media on human development. Experts point out that the reason why users are willing to establish a lasting relationship with a certain social media without transferring to other social media with similar services or content is not only because they do not have the motivation or behavior habits to convert, but because they have emotional attachment. The insight into the formation mechanism of this attachment process is very important for the continuous development of social media platforms (Cao et al., 1987; Teo et al., 2018). However, existing research is still limited in exploring the attachment relationship between users and social media. Therefore, deep analysis of the attachment relationship between users and social media develops in the context of the rapid development of a new round of information technology and the widespread application of local social media. This analysis establishes a continuous connection between information technology products and human development and urges people to recognize the inevitable requirements of how social media promotes human development in multiple fields. Attachment relationship has significant advantages in deciphering the individual differences, intensity, and quality of interpersonal relationships and is the optimal theoretical framework for exploring human intimacy (Hazan and Shaver, 1987). This means that the attachment relationship is of great significance to the deep analysis of the connection between social media and human development.

“Attachment” originated in the field of mother-infant relations, which refers to a strong emotional bond formed between an individual (mainly a baby) and a specific object (mother or caregiver), and it is believed that attachment is a basic human need, which continues throughout the life of the individual (Bowlby, 1969). As a construct based on relational processes, the research and application of attachment theory has expanded from the category of interpersonal relationship to other relationship fields, and it has been well-expanded and applied in the field of personal relationship, for example, research on parent–child attachment, marital attachment between lovers or spouses, and peer attachment in the adult attachment research thematic area, among others (Bretherton and Waters, 1985; Armsden, 1987; Collins and Read, 1994); research on user-brand relationships and brand loyalty in the thematic area of brand attachment research, etc (Bidmon, 2017); studies of place identity and place attachment (in the thematic area of place attachment research) (Williams et al., 1992). It can be seen that attachment exists not only between people, but also between individuals and other non-living entities (such as brands, stores, locations, and virtual spaces), as well as in the field of person relationships (Goel et al., 2011). However, unlike the first three studies, which started early and got a series of fruitful research results, the research on the attachment relationship between individuals and social media started relatively late and remains to be a relatively new concept. This is because the attachment between individuals and social media has become a research hotspot with the rise of the information technology revolution and has attracted attention due to the phenomenon of continuous use of information system. Obviously, the study of attachment between individuals and social media is a frontier trend and has yet been described and measured in a process-based form. Its commercial practice and theoretical development need to be further explored.

This research mainly extends the application of attachment theory to local social media usage scenarios and systematically studies the intermediate processes and mechanisms of the attachment relationship between users and social media. In view of the fact that the relationship between individuals and social media usage contains strong psychological phenomena of individual experience and feeling, quantitative research method from the perspective of relatively static and cross-sectional data is difficult to support the interpretation of the intermediate process, while the qualitative research method seems to be more suitable to explicate the complicated interactive relationship between individuals and social media usage. Compared with quantitative research, the advantage of qualitative research lies in its dynamics to illustrate the development process of psychological events (Tufford and Newman, 2012). Therefore, this study takes the continuous use of social media as a leisure and entertainment as an entry point, to explore the changes in user relationships in the context and its psychological significance to individual users. The study analyzes the intermediate process mechanism of the establishment and development of attachment relationships to find a path to guide user behavior effectively in order to grasp the opportunities and challenges of information technology and promote the active interaction of social media with individuals and the active application in their personal life and work.

This study is innovative in the following aspects. First, this study introduces attachment theory into the context of social media use, expanding the research context of attachment theory. This is a concrete extension and application of attachment theory from physical environment to specific virtual environment of social media, providing a new perspective and useful reference for subsequent studies. Second, this paper reveals the mechanism of social attachment in the context of social media in China, explores the influence of theoretical logic of social attachment on intention of users to consistent use, and expands the relevant research explaining the mechanism of intention of users to consistent use from the perspective of social attachment. Third, this study echoes Zeithaml et al.'s (2006) view that “attachment theory can provide a theoretical guide for customer relationship research, extending the depth and breadth of current marketing research.” Furthermore, the study agrees with the idea that “attachment relationships are seen as an important theoretical framework for explaining human intimacy and have significant advantages in explaining the relationship between individuals and specific objects” (Hazan and Shaver, 1987). Thus, the study has enriched the research on attachment theory. The remainder of the paper is organized as follows. The second section is structured for literature review, and the next section is about research methods. After that, data analysis result is demonstrated, followed by discussion. And the part of conclusion ends the whole paper.

## Literature Review

### Continuous-Use Behavior of Social Media Users

Prior studies have discussed the continuous use of social media from the perspective of information systems. The one of the main viewpoints in this field is the expectation-confirmation model (ECM) proposed by Bhattacherjee (2001), which believes that the continuance intention of users is the subsequent acceptance behavior caused by a series of decisions about whether to continue to use (Gan et al., 2018a). However, long-term use behavior means irrational, but the view of information systems defines continuous use as rational judgment behavior. Because starting from rational characteristics, continuous use requires several independent and unanimous judgments. Obviously, this short-term logic is obviously not applicable to the long-term problem research of continuous use. Relevant empirical research shows that it has an impact on user acceptance and continuous use. The prediction lacks a strong final explanatory power (Zhao and Wang, 2016). In this regard, some studies have conducted research from the perspective of emotional attachment and believe that continuous-use behavior has irrational and long-term characteristics; that is, it violates the cost–benefit logic and is willing to spend more time, energy, money, and other costs to maintain social media long-term use (Zhao and Wang, 2016).

Despite the accumulation of some research results on continuous use and retention of users, many important issues regarding continued use of social media still exist and need to be resolved urgently. In the context of social media use, personal use behavior is mainly voluntary rather than mandatory, and it is relatively easy to switch from one social media to another similar social media, and the transfer cost is low or no cost. Therefore, the commitment of user to continuous use is very important to social media developers. However, in addition to very limited empirical evidence, the problem of continued use of the user has not been resolved in the existing relevant literature.

### The Antecedents of Continuance Intention of Users Toward Social Media

First, social value reflects the needs of users who want to use social media to carry out social activities. Through social activities, users can gain support from others and a sense of belonging (Cho et al., 2014), for example, the effects of factors related to social needs, such as social needs, maintaining relationships, and establishing new relationships, on user behavior (Dermentzi et al., 2016; Jung and Sundar, 2016). The existing literature believes that through the group chat, sharing and private message functions provided by social media, users can meet like-minded friends and maintain relationships with them (Gan et al., 2018b; Chen et al., 2020; Hu et al., 2020; Yang et al., 2020).

Second, users can meet their entertainment value through the diversified functions provided by social media, such as instant video calls, likes, comments, and forwarding messages (Park and Lee, 2014; Ifinedo, 2016). Entertainment needs mean that users want to feel relaxed, fun, and enjoy when participating in social network activities (Dermentzi et al., 2016). Social media exposes users to a variety of sensory stimuli, such as multiple software content, interesting pictures, innovative opinions and discussions, etc. so they are considered by users as a tool for entertainment and communication (Ng, 2016).

Third, information value is defined as the users hope to obtain and share useful information through social media to improve themselves and solve problems (Katz et al., 1973). As a convenient source of information, social media provides users with a variety of information, attracting users to use social media (Chiang, 2015). The existing literature has proposed related factors such as information search and information sharing. Information search refers to users who want to seek useful and helpful information through social media. Through social media, users can track events, trends, music, and other user information (Basak and Calisir, 2015). Therefore, obtaining and sharing information is also considered to be the main motivation for users to use social media.

### Consequences of Continuous Use of Social Media Users

#### First, Customer Satisfaction, and Loyalty

Satisfaction is a comprehensive evaluation of product performance by users, usually extracted from the expectation-confirmation/non-confirmation paradigm (Oliver and Richard, 1980; Prakash, 1984). Its essence reflects the perception of product functions and evaluation by users. In the context of social media use, users first form expectations before use and establish an experience with social media or its functional attributes (such as social functions, entertainment functions, and information functions), then compare the functions with their previous expectations, and finally do make a judgment of satisfaction (Curtis and Tamilla, 2009). However, the impact of satisfaction on users is a short-lived, specific experience, which is affected by the expectations and perceived performance brought to users by social media (Bhattacherjee, 2001). Therefore, when there are better-functional social media alternatives, satisfied users may switch to alternative social media. Especially when the homogeneity competition of social media continues to intensify, a factor more “sustaining” than satisfaction is needed to affect users of social media. In this regard, some researchers believe that user loyalty is more important than satisfaction, because it is of strategic importance to social media that is interested in obtaining sustainable competitive advantages (Gounaris et al., 2003).

Loyalty refers to “a long-term commitment to consistently re-use social media in the future, although it may be affected by unexpected circumstances and lead to conversion behavior.” Its essence reflects a powerful emotional power of users toward the product. Reichheld and Schefter (2000) believe that loyalty not only costs less to retain an existing user than to acquire a new user, but also that loyal users spend more than disloyal users and participate in additional services more often. Reichheld and Schefter (2000) state that when a user is more satisfied with alternative social media due to superior performance or better system task suitability, he may move to alternative social media. On the contrary, loyal users are less likely to transfer. For example, a loyal user who has seen innovative features in the competition would rather integrate these features into the social media he is using, rather than switch to social media of a competitor, because he would rather improve his loyal social media (Zhao and Wang, 2016).

#### Second, User Immersion

Immersion refers to a subjective state in which the individual is engaged in the situation and filtered out irrelevant perceptions when he is performing certain daily activities, thereby focusing his attention on the current activity (Csikszentmihalyi and Lebuda, 2017). Immersion is mainly reflected in perceived enjoyment, perceived practicality, perceived control, as well as concentration and time distortion.

Perceived enjoyment refers to “a kind of subjective pleasure generated by individuals in the process of using social media, which reflects users that are happily immersed in it and enjoy the virtual world constructed by virtual scenes to achieve a pleasant experience of body and mind integration” (Li, 2017). Perceived practicality refers to the result of users consciously pursuing the intended purpose. It has the characteristics of instrumentality, functionality, and recognition. When users think that social media is useful, they will conduct exploratory browsing and search for relevant and useful information as needed information, etc., may lead to immersion (Chien et al., 2012).

Perceived control is described as a kind of perception when a person feels that they are in control of their behavior and the interaction with their environment (Fave and Massimini, 2005). In the context of using social media, users can control their behaviors such as like, comment, and forward. When users have control over social media, their behavior will be more active [35]. Time distortion refers to the phenomenon that individuals lose their sense of time. Through beautiful interface design, powerful function design, and sensory stimulation, social media allows users to immerse themselves in it unconsciously and have a distorted cognitive feeling about time. Under these circumstances, users focus on the social media situation rather than get rid of it, thus forming an addictive state (Xie and Zhu, 2019).

The above-mentioned research on the attachment relationship of social media users expands the research object of attachment relationship and extends the previous research on mass users and social media to the study of the relationship between users and social media, laying a foundation for the in-depth study of social media user behavior basis. However, the existing research still has the following shortcomings: One is the lack of research on the intermediate process and results of the attachment relationship between users and social media. From previous studies on social media usage behavior, users continue to use social media because they are stimulated by social value, entertainment value, and functional value. Then, how do users generate these values? The causes and consequences of this lack further discussion. The second is the attachment relationship between users and social media. What kind of result is produced, whether it is user loyalty or user immersion, still lacks a clear conclusion and needs further discussion.

## Research Methods

This research mainly uses the grounded theory in the qualitative study method to analyze the attachment relationship between individuals and social media. According to the grounded theory, selecting representative research objects for in-depth research and constructing new theories or updating theories is a qualitative research method proposed by Charmaz (2009). That is, a scientific method of qualitative research on a specific phenomenon is established through rooted, theoretical, and constructive system of encoding, summarizing, refining, and abstracting the original data. This article is based on the continuous-use intention and behavior phenomenon of social media users, selects representative and relevant local social media as the data source, and conducts an in-depth analysis of the data. Try to discover and reveal the purpose and mechanism of continuous-use behavior of social media users and their impact on social media relationships. This is a typical generation process from phenomenon to theory, suitable for grounded theory.

### Samples

This study selects three social media platforms, namely, TikTok, WeChat, and MicroBlog as sources of collecting sample data. The reasons are as follows. First, these three local social media platforms belong to different subcategories and occupy the largest population of users. Currently, TikTok has 600 million daily active users, WeChat has 1.09 billion users every day, and MicroBlog has 5.11 million monthly active users. TikTok is a short video APP. WeChat is a graphic information APP, and MicroBlog is a traditional APP with a certain degree of breadth in the distribution of social media. Second, TikTok, WeChat, and MicroBlog are all locally pioneered social media, and they take leading positions in their respective market segments. Among them, TikTok is a fast-growing company. Since its inception in 2012, it has established itself in the local social media platform, occupying the first place in the market share. Therefore, these three types of social media have the typical characteristics required by the case study. Third, all the above three have gathered a large number of users in the process of enterprise development and possessed the representative characteristics required by qualitative research.

### Data Collection

#### Pre-interview

Before the in-depth interview, the researcher used a comprehensive sampling strategy to select seven senior TikTok players for a pilot pre-interview. The pre-interview centered on the research theme of “brushing TikTok” and invited the interviewees to make free associations based on their own understanding and ideas of TikTok, and report on their behavior and inner change process as a senior user, so as to understand and grasp the experience and inner feelings of an interviewee.

The structure of the pre-interview is relatively weak. The researcher only plays a supporting role in the pre-interview, encouraging the interviewees to express their opinions in their own language, in order to understand what it means to use TikTok in the eyes of the interviewee. What do they think is the most important question about “brushing TikTok?” What is their perspective on the problem, the concepts they use, and how they are expressed? Provide a basis for the next step to design a formal interview outline.

#### Formal Interview

To further ensure that the interviewee is our target audience, we used two filtering questions at the beginning of the formal interview to check the qualifications of the participants. That is, one is to exclude users under the age of 18, and the other is to exclude users with <6 months of the target software using experience.

The actual interview focuses on three questions. The first is the general usage behavior of the social media users (their habits of using social media, the contents of their interest, their inner experience, etc.). The second is their experience of using social media (such as sharing their daily activities, meeting friends, and learning various anecdote Gonzos). The third is the relationship between users and the relationship between users and social media in the process of brushing (i.e., using) social media (such as changes in emotional bonds between users and the emotions that users have on social media itself). These three questions are of a gradual and in-depth relationship. The general situation of swiping of social media users covers a wide range; the experience of using social media has begun to focus on the behavior of social media brushing, to the theme of attachment relationship being further extracted. In the end, when the information provided by the interviewee in the interview reached or basically reached saturation, the two sides jointly decided to end the interview. On this basis, a following-up research was conducted with users of the above three social media from March to December 2019, and a total of 27 users as data collection objects had been interviewed on their experience on the three selected social media. After the interview records of these 27 users are classified and processed, the data are cleaned to form a word file, which became the source material that NVivo12 can import. The demographic characteristics of the in-depth interview samples are shown in Table 1.

**Table 1:** Demographic characteristics of in-depth interview samples.

**Data Sources**	**User ID**	**Gender**	**Age**	**Educational Background**	**Profession**	**Cumulative use time**
TikTok	xiangcunxiaolei	Male	25	Bachelor	Country startup	Over 3 years
	Heyuan0617	Female	22	Bachelor	Clerk in private enterprise	1–2 years (inclusive)
	Syxlb123	Male	35	High school	e-Commerce startup	6 months to 1 year (inclusive)
	Xiangyi1616	Female	27	Doctoral candidate	Student	1–2 years (inclusive)
	YS178728	Female	28	High school	Housewife	1–2 years (inclusive)
	Qiangzi202001	Female	30	Junior college	TikTok startup	1–2 years (inclusive)
	2211832341	Male	30	Junior college	Executive in private enterprise	1–2 years (inclusive)
	183726223	Female	26	Bachelor	Airhostess	1–2 years (inclusive)
	Pu0802	Female	30	Bachelor	Startup	Over 3 years
	Abby20170130	Female	30	Junior college	Housewife	Over 3 years
	Thingdifferent99	Male	45	Junior college	Startup	1–2 years (inclusive)
MicroBlog	Yuyuanbo1205	Female	33	Bachelor	High school teacher	Over 5 years
	Chen Shiyun	Male	26	Doctoral candidate	Student	Over 10 years
	Mak	Female	26	Bachelor	Writer	Over 5 years
	Fireworks	Female	20	Bachelor	Clerk in state owned enterprise	Over 5 years
	Red Maple	Female	23	Bachelor	Freelance	Over 5 years
	Little Fairy	Female	29	Junior college	Civil servant	7–10 years (inclusive)
	Redika	Female	23	Bachelor	freelance	7–10 years (inclusive)
WeChat	Uruhr_aqz	Female	20	Bachelor	Editor	7–10 years (inclusive)
	Jerryyf1985	Male	35	Bachelor	Manager of private enterprise	Over 10 years
	Wxid_sjc1ypgp21	Male	35	Bachelor	Executive in private enterprise	Over 10 years
	Zj8843666	Male	37	Junior college	Startup	Over 10 years
	Wuxueshi0047538	Male	35	Doctoral candidate	College teacher	Over 10 years
	A Youngboy	Male	30	Junior college	Executive in private enterprise	7–10 years (inclusive)
	Aowang112	Female	38	High school	Startup	Over 10 years
	Zhengwei5070	Male	40	Doctoral candidate	College teacher	7–10 years (inclusive)
	Yh512399184	Male	26	Junior college	Executive in private enterprise	7–10 years (inclusive)

### Data Analysis Method

Based on grounded theory, NVivo 12.0, a representative tool of qualitative analysis, is used to analyze the text data from bottom-up. By importing interview texts, the software indexes, searches, and theorizes unstructured and non-numerical data, which is helpful for researchers to code and search, establish indexes, logical relations, and theories, etc. (Berends and Deken, 2021). The whole process is orderly promoted under the guidance of relevant expert groups. After discussion, a theoretical model of the intermediate process and results of continuous use of social media users is proposed.

## Data Analysis

### Initial Coding

Initial coding refers to the step-by-step decomposition of the original interview data, from which points and sentences of the smallest meaning unit are identified, and any recorded data that can be coded are given a conceptual label based on the existing literature (Charmaz, 2009). In this study, in order to ensure the rigor of the research process and the scientific nature of the research conclusions, two groups of researchers separately coded the collected data from the interview to ensure the reliability of the initial coding (Chen, 2000). The tags are sorted repeatedly. And finally, the initial coding system is concluded.

First, before the actual analysis, set a corresponding number for original interview data of each respondent (such as TikTok7, MicroBlog7.WeChat7), in order to prevent confusion among the interviewed data provided by interviewees in the follow-up data analysis process, which lays the foundation for data analysis. Second, in the initial coding stage, the coder uses NVivo12 to analyze the verbatim content, sentence, and paragraph in the original data imported into the system, taking “the attachment relationship between the user and the social media in the process of individual interaction with social media” as core (Glaser and Strauss, 1967; Charmaz, 2009; Chen, 2015), to look for the recurring meaning units from the data, to extract the login code that is meaningful to the research topic, and to encode the data. Third, the research results obtained by the two groups of researchers are discussed. Following the principle of the same or similar meaning, the coding results of the two groups of researchers are sorted and summarized. The concepts with less frequency are eliminated, and the repeated or similar concepts are merged. Then researchers check the coding results and make a decision through literature analysis and consultation with relevant experts on the discrepancies in the coding results. As a result, a total of 681 references are extracted and 82 nodes are constructed. Furthermore, according to the number of references contained in each node, all nodes are unified into the coding system in the order from more to less to form an initial coding system.

### Focused Coding

Focused coding is based on the initial coding; according to the paradigm of “condition → action/interaction strategy → results,” the initial coding is clustered and analyzed and arranged and combined in a new way to form a new and general category (Berends and Deken, 2021), which is focused coding. Focused coding reflects the internal interconnection of different categories obtained in the initial coding at the conceptual level, as well as the relationship structure between the main category and the subcategory (Strauss and Corbin, 1998).

Since the 82 nodes extracted from the initial coding present different meaning units, further sorting and analyses are needed. Through the analyses of existing categories and the mining and comparison of original interview data, NVivo12 software is used to further refine the initial coding system around “the attachment relationship between the user and the social media during the interaction between the individual and the social media” (Kuhn, 1962; Charmaz, 2009; Chen, 2015); the 82 nodes are summarized and refined once more, and new themes emerged and developed from the interview data. As a result, 14 nodes related to the research theme are obtained. Specifically, the researchers classify “intentional participation,” “information sharing,” and “interpersonal interaction” into the same focus code, namely, user participation. They treat the three code numbers of “social function,” “entertainment function,” and “information function” as the same focal code, that is, co-creating value. They make heterogeneous comparisons by clustering “identity construction” and “emotional expression” into the same focus code, namely, “self-expression,” and refine “relationship needs,” “ability needs,” and “independent needs” into the same focus code, that is, “needs satisfaction.”

### Axial Coding

Axial coding is adopted to select a core category after systematic analysis on the basis of the conceptual category from focus coding. The core category must be repeatedly proven to be commanding in comparison with other categories and must be able to include most of the research results in a relatively broad theoretical range (Charmaz, 2009). Axial coding aims to focus on the categories formed by the coding, to continuously develop and link the gradually emerging relationships between categories, and to conceptualize the association structure between categories through coherent stories (Charmaz, 2009). The genus relationship formed by focused coding is further concretized, a story line that can dominate the entire category is developed, and a theoretical model is formed based on all relationship conditions and behavior phenomena described by the story line (Csikszentmihalyi and Lebuda, 2017).

Through further analyses of the main categories formed by focused coding, this study has five groups of codes connected by the “story line” and the connection between the categories is established. The intermediate process mechanism of the relationship between individuals and social media is analyzed to form a story line that dominates the entire category. Specifically, researchers make a longitudinal comparison of the original data provided by the same interviewee and make a horizontal comparison of the original data provided by different interviewees. It is found that the four codes, namely, “user participation,” “co-creation of value,” “self-display,” and “psychological needs,” reflect three levels of attachment relationships. Therefore, these four codes are classified into three different categories. The re-clustered focused coding is logically analyzed according to the story line. The results show that individuals stimulated co-creation value through participation behaviors. In the context of individual continuous use, co-creation value forms an attachment relationship through the mediating effects of self-display and psychological needs (see Table 2).

**Table 2:** The formation of axis coding and its theoretical system.

**Nodes**	**Focused coding**	**Axial coding**	**Connotation of axis coding**	**Theoretical system**
81 73 67	Interested in participating Information sharing Interpersonal interaction	User participation	User participation refers to the exchanges, interactions, and activities between users and social media and other users.	User participation creates continuous-use scenarios for attachment relationships, which is the prerequisite for the attachment relationship to occur.
57 56 47	Social function Entertainment functions Information function	Co-creation value	Co-creation value refers to the active co-creation of value of users with social media and other users in the context of using social media.	Co-creation provides a driving role for the occurrence of attachment relationships, which is the source of stimulation for attachment relationships.
42 36	Identity construction Emotional expression	Self-expression	Self-presentation means that users communicate some information about themselves to others through social media, so as to gain respect or status.	Self-expression provides mediating effects for the occurrence of attachment relationships, which are mediating effects of attachment relationships.
45 37 33	Relatedness satisfaction Competence satisfaction Autonomy satisfaction	Psychological needs	Psychological needs are the response of users to individual needs through social media, so that users can gain a sense of emotional and psychological security.	Psychological needs provide mediating effects for the occurrence of attachment relationships, which are mediating effects of attachment relationships.
39 34 35	Social connection Social dependence Social identity	Social attachment	Social attachment refers to an emotional and unique bond between the users and local social media.	Social attachment is the result of the attachment relationship between individuals and social media, which is the behavioral response that occurs in the attachment relationship.

### Theory Construction

Based on the grounded theory, the present research constructs the theory bottom-up from the data, analyzes the data through the coding and coding process, integrates the concepts and types discovered from the data, and gradually generates the theory in the inductive analysis (Charmaz, 2009; Chen, 2015). Four steps are shown as follows. First, core categories or topics on the basis of three-level coding are further explored. On the nodes established by the three-level coding, the relationship between different categories is further analyzed according to the characteristics of the data, and the four categories, namely, “user participation,” “co-creation value,” “self-expression,” and “psychological needs,” are found. Each node contains or is covered by the “social attachment” node, and then, the “core category,” that is, “social attachment,” is gradually produced in the inductive analysis.

Second, a theoretical framework is established for the formation and development of social attachment based on the “story line.” Along the story line of the formation of social attachment, five groups of nodes generated by the three-level coding are restored to the interview data of the interviewees. The analysis finds that the co-creation value of an individual has a social impact through the mediating effects of self-expression and psychological needs. Attachment has a positive effect. In order to further verify and refine the results of the three-level coding, an in-depth analysis is conducted and deduction of the themes is selected and formed by the axis coding, and finally, the theoretical analysis framework of social attachment is extracted, and the initially established theoretical framework is restored to the original interview data. Through the comparison and verification of the original data, the theoretical model is finally proposed after discussion.

The third step is to establish a theoretical system with internal consistency. Focusing on the “attachment relationship between users and social media in the process of interaction between individuals and social media,” a theoretical system is constructed along the story line: The co-creation value in the context of social media use provides a source of stimulus for continuous-use behavior of the individual, changes the individual self-expression and psychological needs, and promotes the continuous development of the attachment relationship, which leads to the emergence of social attachment. Among them, user participation is a prerequisite for driving the occurrence of co-creation value, creating continuous-use scenarios for the interaction between individuals and social media; psychological needs and self-expression play an intermediary role between co-creation value and social attachment and promote the occurrence of sense of belonging of an individual to social media.

**Figure 1:**
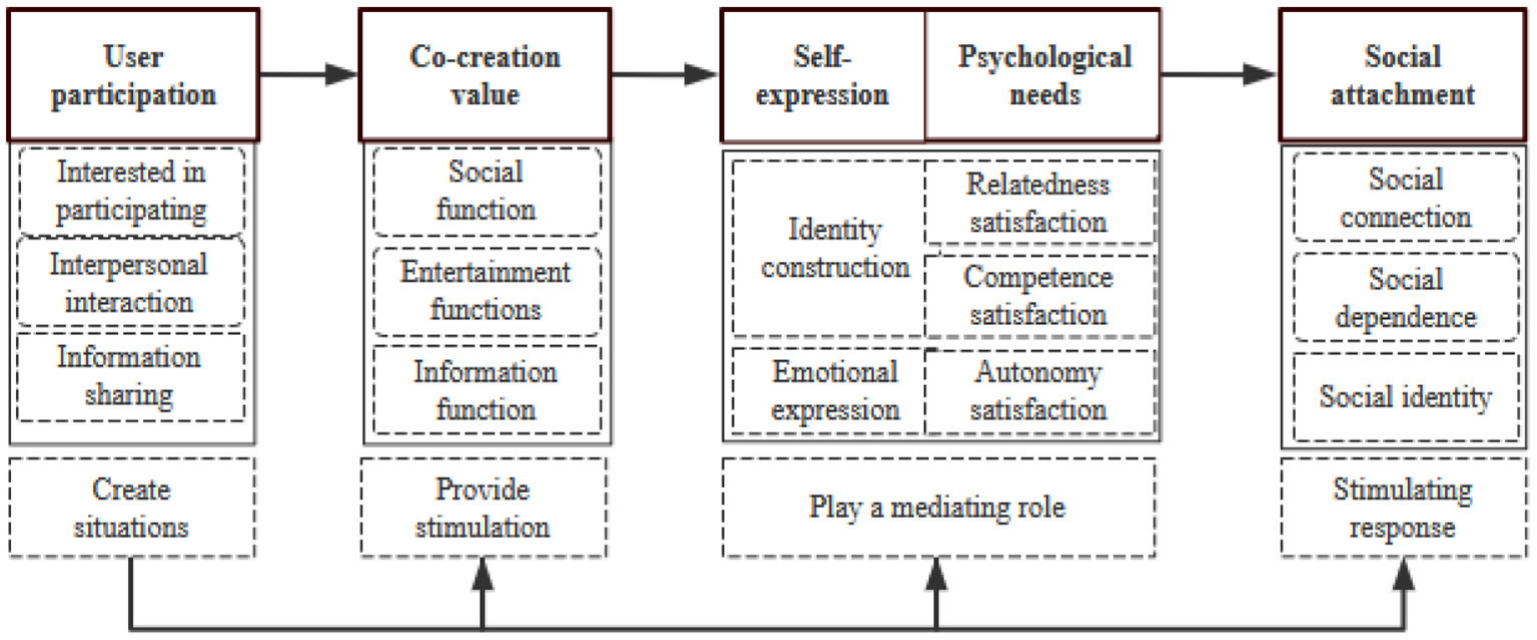
The mechanism of social attachment.

Fourth, the degree of saturation of the theoretical construction is tested. Data collection and analysis run through the entire research process until the “theoretical saturation” is reached. In this study, the sampling is stopped until there is no new content after the 21 consecutive samples are drawn. In the process of theoretical construction and repeated verification of data, this study randomly selected nine participants from March 15 to May 30, 2020, for saturation test, and fed back the coding and research conclusions to interviewees, and conducted data analysis to circulate verification and testing and to compare and analyze with existing theoretical models in order to improve the validity and quality of the research process and conclusions (Wu and Huang, 2012). According to the feedback of respondents on the coding and results, no new concepts, categories, or relationships have been extracted. Based on the above analysis, it can be considered that the model has reached theoretical saturation. The study extracts the connotation of the interaction between individuals and social media along the story line and then clarifies the mechanism and results of the continuous behavior of social media users (Figure 1).

## Discussion

### Co-creation Value Creating Software Using Context

The essence of user involvement in social media platforms is a process wherein values are created. As a result of participation behavior of users, three categories of values are created, namely, social value, entertainment value, and information value. According to the story line formed by the qualitative research, it suggests that user perception of the value of using social media enhances their continuous use and even immersion with the social media. Therefore, in the context of social media usage, user participation can increase their perceived benefits and value. From the point of view of users, co-creation value may be originated from the user participation in meeting the needs of an individual or benefited from the relationship between the individual and others (Park and Lee, 2014).

First, user participation will actively initiate “interesting” interactions, use vivid and lively language to publish extremely ridiculous and hilarious interactive contents, give fans a pleasant emotional experience, and then form a positive attitude toward the social media (Ifinedo, 2016). Therefore, the interpersonal interaction between users has a significant positive impact on co-creation value.

Second, sharing information and opinions is always interactive [23]. The more involved users get in the interaction, the more value of attaining the common sense of life, product knowledge, and the useful skills is created from the continuous usage of social media platforms. As a result, users are more motivated to develop their attachment to the social media. Therefore, user sharing of information has a significant positive impact on co-creation value.

Third, users are willing to expand their relationship through social media platforms; socialize with people of common interests, goals, or needs; interact with each other; share information; and communicate with group members. This enables them to develop a sense of belonging and identification with online community. In turn, they may also have an identification with the social media platforms and strengthen the connections and tacit understandings about social media platforms, forming a relatively stable social value, entertainment value, and information value. Therefore, an intentional participation of users has a significant positive impact on co-creation value.

### Co-creation Value Stimulating Self-Expression

First, users construct virtual identities through co-creation value. The individual virtual identities in the context of social media use refer to the virtual identity constructed by an individual through the social media platforms, and the self is constructed, displayed, and expressed with freely expandable symbolic tools (text, images, short videos), etc., which reflects the virtual identity that social media users want to express and show to others. In the process of identity construction, users display themselves through co-creation value, which is generated by the conscious participation and interpersonal interaction of the users. For example, TikTok users engage in social activities by sharing life activities and learning about interesting events, create co-creating social value and entertainment value together, and construct virtual identity in co-creating value.

*For example, the interviewee Tik Tok 7 expressed this view: Share the bits and pieces of travel on the TikTok, show the scenery of nature with photos and videos, and communicate with more friends in the form of travel diaries. As the number of contacts will increase, The emotions between us will be stronger. In the process of implementation, we received very expected responses and role support in the interaction through short videos, as well as many likes, comments and reposts from strange friends (co-creation value*→*virtual identities)*.

Second, users express emotions through co-creating value. With the advent of the era of big data and the rapid development, maturity, and popularization of social media, social platforms such as TikTok, WeChat, and MicroBlog have become the main places for people to socialize and entertain. In the context of using social media, individuals can publish and share their current state and emotions anytime and anywhere on the social media platforms, as well as expressing their pressure and exchanging their joy.

*For example, the interviewee A-6-22-2 expressed this view: I share information and opinions on postgraduate entrance examination knowledge on MicroBlog every day, such as English grammar, beautiful short sentences, vocabulary and composition, and record my postgraduate entrance examination life on MicroBlog. Looking at my own growth experience is also an encouragement for me. At the same time, you can also learn about the postgraduate entrance examination status between friends and form healthy competition and progress. In short, I think this is good (co-creation value*→*express emotions)*.

Social media has gradually become an important channel for people to express their psychological state. On the typical social media platforms such as TikTok, WeChat, and Weibo, they record their daily life; share information and interact with friends; express their happiness, anger, fear, sadness, surprise, aversion, and other emotions; relieve stress and depression; and get satisfaction and happiness in virtual reality.

### Co-creation Value Meeting Psychological Needs

In the context of using social media, users respond to the basic psychological needs of individuals through the co-creating value process of information function, and they obtain a sense of emotional and psychological security. They have a sense of belonging to the social media, so that the needs of users can be continuously met. This kind of psychological needs is met through the following three ways.

First, under the stimulation of co-creating value, users need the care, understanding, and support from the surrounding environment or others, hoping to belong to a certain social group and form a close relationship with others. Through co-creating value, users can satisfy their relationship needs on the social media platform.

*For example, the interviewee Tik Tok-2019-6-6 expressed this view: Yesterday, a short video of mine went viral. It was less than an hour after it was published. The number of views exceeded 3 million, the number of likes exceeded 677,000, and the number of comments was 47,000. It is still rising. In the comment area, a lot of fans asked me how to break the broadcast, whether there are any shortcuts to the copywriting, etc. (opinion leaders-ability required). At first, I responded one by one, but then I found that I was too busy to respond to all the comments in the comment area. Because I couldn't respond at once, I left a message at the top of the comment area “Every morning at 5:40, share the broken broadcast in the live broadcast room (*co-creation value → relationship needs*)”*.

Second, under the stimulation of co-creating value, users experience a sense of competence in their interactive activities with social media, and meet the pursuit of efficiency and achievement, so as to achieve the satisfaction of capacity needs.

*For example, the interviewee Tik Tok-2019-8-1 expressed this view: There were 17 live broadcasts, and the number of people in the live broadcast room has not been able to come up. It really makes people break down and lose confidence. I even started to doubt myself. Anyway, I am also a college student and I am not ugly. Why is there no popularity? So I didn't control myself and burst into tears in the live broadcast room, feeling very wronged. At this time, a lot of fans came to comfort me, and they left me a message saying that they support me and let me cheer (to get comfort/emotional support-the relationship needs to be satisfied). It is precisely because of the support of the fans who I have not met before that I have today's results. I have persisted in the live broadcast, and now I have begun to bring goods (*co-creation value → capacity needs*)*.

Third, under the stimulation of co-creating value, users can choose their behaviors freely according to their personal needs and the evaluation of environmental information, and the extent of the self-selection and self-control of an individual when facing the pressure of external events that affect individual behavior, so as to achieve the satisfaction of autonomous needs.

*For example, the interviewee Tik Tok-2019-7 expressed this view: Now, I use TikTok almost every day. If I don't do it, I feel uncomfortable. I can upload my short videos on TikTok to share the moments in my life. Yesterday I also uploaded an egg soup made in the kitchen. Now I have a lot of likes and comments, and many people want me to WeChat (*co-creation value → autonomous needs*)*.

## Conclusions

### Research Results

#### Revealing the Mechanism of the Attachment Relationship

Based on the grounded theory as a guide, this research adopts the paradigm of qualitative research, to find the mechanism model of user behavior of social software in China. It points out that user participation creates usage scenarios for the interaction between individuals and social software, in which co-creation value is formed driven by user participation; co-creation value elements act as stimuli to stimulate the user emotional and cognitive state of social software, so as to stimulate psychological needs and self-expression of users, and promote and strengthen sense of belonging of users to social software. Psychological needs and self-expressions are used as intermediary variables to influence approach of users to social software and to promote the formation of social attachment relationships between users and social software. After the formation of social attachment, it dominates the attitude and behavior tendency of users, while the original cost-benefit logic and cognitive judgment of users take a back seat, which in turn continues to strengthen the interaction between users and social software, creating a context for continuous use of social software by users.

The mechanism of social attachment formation conforms to the stimulus-organism-response (S-O-R) theory (Mehrabian and Russell, 1974). In this research, user participation creates preconditions for the establishment and development of social attachment relationships. Co-creation value is the antecedent variable (stimulus), psychological needs, and self-expression are the mediating variables (organism), and social attachment is the result variable (response). Therefore, in the context of continuous use of social software, the user is stimulated by co-creating value (S), and the internal state (O) of self-expression and psychological needs have changed, which leads to the production of behavioral response (R), that is, social attachment.

Social attachment is an attitude model that includes cognition, emotion, and behavioral intention. In the context of social software use, users participate in activities through social software platforms and then produce a lasting emotional connection, in which users tend to seek and maintain a close relationship with social software. Especially when psychological needs of users are met and self-expression is expressed, social software users form, and gradually deepen their sense of efficiency, accomplishment, and novelty. Gradually, quantitative changes cause qualitative changes, the cognitive system of social software users is reorganized, and the realization of psychological needs is combined with social software to form attachment relationship. At the same time, when they need to show themselves, they feel the support and comfort provided by social software during their use, which makes social software the object of attachment of users, and forms a long-term and stable attachment relationship between individuals and social software.

In this paper, qualitative research method is used to analyze and summarize the formation and development of attachment relationship strictly from the interview data, redefine the relationship between users and social software, reveal the intermediate process and results of the mechanism of local social software users, and make up for the insufficiency of the previous literature on the mechanism of social software user behavior. The discussion of the main issues such as the continuous development of local social software and attachment relationship provides knowledge basis and theoretical guidance for the follow-up academic research, industry practice, and the formulation of industry rules by the government.

#### Discovering the Intermediate Mechanism of the Attachment Relationship

User participation creates scenarios and situations in which social attachment systems occur. The research results show that with the continuous progress of user participation, the gradual change in the relationship between users and social software is as follows: co-creating value → self-expression/psychological needs → social attachment. This relationship between the two parties is a manifestation of attachment relationship, because as long as a long-term emotional connection is formed between an individual and a specific object, it means that attachment is formed and exists in a subjective and systematic way within the individual (Bowlby, 1982). From the research of this article, social attachment of users is formed through two ways.

First, in the context of using social software, co-creation value of users has a positive impact on social attachment through self-expression. Co-creation value is a predictive factor for self-expression, indicating that users have a strong demand for self-identity construction and emotional expression. Self-expression is “using behavior to convey some information about oneself to others” (Baumeister, 1982). These positive effects brought about by self-expression can enhance the status and satisfaction of social software in the minds of users, thus forming a strong sense of belonging and then a strong attachment to social software. In this research, users awaken self-expression through co-creation value, which stimulates users to construct virtual identities and express emotions, thereby forming social attachments. Therefore, social software provides users with a wider range of interaction opportunities and becomes a promoter of co-creating value, which greatly stimulates self-expression. Through the mediating effect of self-expression, an attachment relationship has occurred between users and social software.

Second, through co-creating value, users obtain basic psychological needs, which has a positive effect on social attachment indirectly. According to the self-determination theory, the basic psychological needs of individuals include autonomous needs, ability needs, and relationship needs. In the context of using social software, user experience of intimate or sincere contact with others on the social software platform (Ryan and Deci, 2000) enhances continuous satisfaction of relationship needs and effectively resists frustration, relationship rejection, and loneliness. The use of social software brings users a sense of effectiveness and the satisfaction of achieving expected results so that the ability needs can be met continuously (Nguyen et al., 2018), thereby reducing the feeling of failure resulting from ability setbacks and doubts about effectiveness of an individual. In the interaction with social software, user experience of self-determination, complete will, and determination satisfies the autonomy needs continuously, thereby effectively resisting external pressure or self-imposed pressure and then controlling own emotions of an individual. From the perspective of this research, users meet their basic psychological needs through co-creating value. The basic process from demand satisfaction to attachment emotion formation conforms to the internal working model of attachment formation. The continuous satisfaction of individual needs by social software will promote the cognitive reorganization of individuals and develop emotional attachment.

From the perspective of action path, new cognitive judgments of users on social software and the continuous accumulation of experience such as intimacy, belonging, and support will lead to qualitative changes, so that the cognitive system will be reorganized; the psychological needs and self-expression of the self will be combined with the social software connection, leading to the formation of social attachment. From the above analysis, users meet their psychological needs (self-display and psychological appeal) through the continuous use of social software, thus forming social attachment.

#### Proposing a New Construct “Social Attachment”

Based on the relevant terms of attachment theory (Bowlby, 1982), this study defines the attachment relationship between users and social software as “social attachment,” in which the emotional connection between users and social software is a manifestation of attachment relationships, because as long as a long-term emotional connection is formed between an individual and a specific object, attachment can be formed, which exists in a subjective and systematic way within the individual (Bowlby, 1982). Therefore, according to the expansion and application of the attachment theory in brand field, local field, and adult field, this paper defines “this unique emotional bond between users and social software” as social attachment and refines three dimensions of social attachment: social connection, social dependence, and social identity.

Social connection is a sense of belonging or membership to a group of people (such as friends in WeChat Moments, fans on TikTok, etc.), as well as emotional connections based on shared experiences, interests, or concerns. Specifically, social connection provides scenarios for the occurrence of interpersonal interaction, virtual reality, and cultural relationships. The sense of belonging develops in this interactive relationship shared with others, and the motivation to keep close to the social software platform is also maintained in this interactive relationship. As scholars Mesch and Manor (1998) observed in their research, those who have more intimate friends on the social software platform have stronger attachments to social software.

Social dependence is the suitability of a specific virtual environment to meet the functional needs and specific goals of an individual, which reflects the preference of individual for ideal social software. In the context of using social software, social dependence is a material attachment. The functional dependence of users on a certain social software or a certain feature of social software is investigated, which shows that social software and its functions have specific conditions to meet the basic needs of users. As a form of social attachment, social dependence is a functional connection based on the physical environment. It is not only related to the potential ability of specific functions to meet personal needs and goals, but also related to the comparison and evaluation between current social software and other social software that can meet the same needs, reflecting the degree to which specific physical environmental conditions meet the expected purpose.

Social identity is one of the constituent elements of self-identity of an individual, reflecting the connection between the self and a specific virtual environment based on direct or indirect experience. It is a spiritual attachment used to explain the personal identity of an individual related to the physical environment of social software. It is the synthesis of the feelings and symbolic connections of a specific physical environment, which determines who the user is, and includes the emotional meaning formed by causal expression and self-confirmation. Social identity reflects the degree to which an individual feels connected with and belongs to the social software. Individuals regard social software as a key element of self-expression and identity. By strengthening the connection between self and social software, individuals can enhance their perception of the ability to symbolize the value of social software, and they then achieve a significant positive impact on the continued use of social software.

Attachment is a strong emotional connection between an individual and a specific object in life (Liang and Wang, 2014). From the research of this article, this attachment relationship is reflected in two aspects: One is that the emotional bond formed by the co-creation value of users through psychological needs has a specific impact on the usage behavior of users; the other is that the self-expression of users formed by the co-creation value produces irrational use of social software. Specifically, in the context of social software usage, user participation is a prerequisite for driving the occurrence of co-creation value, creating a continuous-use scenario for the attachment relationship between individuals and social software; user participation drives the occurrence of co-creation value and stimulates the continuous development of social attachment relationships; co-creation value increases the time and energy (stickiness) that users pay attention to social software through psychological needs and self-expression, which are effective ways to form attachments through continuous usage.

### Practical Implications

#### Creating an Individual Continuous-Use Situation Is a Prerequisite for the Formation of Social Attachment

From the research in this paper, outstanding design can make social software stand out and help attract users and gain recognition. The appearance design of social software (for example, elegant appearance, a sense of youth, and innovation) influences the initial impression and serves as a means of self-expression, experiencing intimacy, and developing emotional connections with APP. For users, the use of well-designed social software can inspire the sense of pleasure, psychological freedom, and will of an individual.

Aesthetic design refers to the appearance and beauty of a product (Bloch et al., 2011). Its essence is the differentiated use attributes of the product itself, which can create user-preferred use situations and influence the continuous-use intention and behavior of an individual. Therefore, product design can be created from the perspective of consumers (Reber et al., 2004). Based on this, for social software, the aesthetic design can be continuously optimized to create an individual continuous-use situation.

#### Stimulating Individual Co-creation of Value Is the Stimulating Element for the Formation of Social Attachment

From the research of this paper, entertainment value is mainly derived from a pleasant experience and feeling of the interest, originality, and funniness of the communication contents, which is directly related to personal emotions and moods. Entertainment function is formed in the process of interaction and communication between individuals and other users or platform content through social software platform, which can make the individual feel relaxed, comfortable, and even spiritual pleasure in the interaction.

Functional design refers to the performance of a product or the ability to meet its utility performance (Bloch, 1995). Its essence is to design the function of a product from the perspective of consumer demand, so that its function or service can meet the demand of consumers for aesthetic satisfaction or practical purpose (Batra and Ahtola, 1991), which is also supported by Luchs and Swan (2011). Based on this, for social software, continuous improvement of function design can be used to stimulate individuals to create value together.

#### Shaping Individual Emotional Bonds Is an Effective Way to Form Social Attachment

From the research of this article, there is a significant positive relationship between the symbolic attributes of social software and self-expression and psychological needs of an individual. The more self-image of users can social software express, maintain, and explain, the more needs of individual uniqueness and relevance can be met (Robertson et al., 2019). Therefore, actively constructing the symbolic attributes of product design can improve the demand of an individual for ability and affinity, affect self-expression of consumers, increase the download rate of social software, affect the intention and behavior of continuous use of an individual, and shape word-of-mouth recommendation and marketing promotion. It also affects market share (Ladik et al., 2015) and enhances the sense of belonging and attachment of an individual.

Symbolic design refers to the perceived information about the self-image of the consumer that the product transmits to consumers and others (Ladik et al., 2015; Jindal et al., 2016). Individuals will have emotional reactions to social software with obvious symbol design, because the symbol design not only enhances basic psychological needs and sense of belonging of users, but also allows users to use social software platforms to express and show themselves. Based on this, for social software, it is possible to build individual emotional bonds through continuous construction of symbol design.

## Data Availability Statement

The original contributions presented in the study are included in the article/Supplementary Material, further inquiries can be directed to the corresponding authors.

## Ethics Statement

Informed consent was obtained from all subjects involved in this study.

## Author Contributions

MY and WZ involved in conceptualization and involved in writing the review and editing. MY, AR, and YZ involved in data curation. MY and AR performed formal analysis. MY, WZ, and YZ performed investigation. MY, WZ, and AR involved in writing the original draft. All authors have read and agreed to the published version of this manuscript.

## Conflict of Interest

The authors declare that the research was conducted in the absence of any commercial or financial relationships that could be construed as a potential conflict of interest.

## Publisher's Note

All claims expressed in this article are solely those of the authors and do not necessarily represent those of their affiliated organizations, or those of the publisher, the editors and the reviewers. Any product that may be evaluated in this article, or claim that may be made by its manufacturer, is not guaranteed or endorsed by the publisher.
